# Patient safety culture, missed Nursing care and its reasons in Obstetrics*

**DOI:** 10.1590/1518-8345.4855.3461

**Published:** 2021-06-28

**Authors:** Silvana Cruz da Silva, Bruna Xavier Morais, Oclaris Lopes Munhoz, Juliana Dal Ongaro, Janete de Souza Urbanetto, Tânia Solange Bosi de Souza Magnago

**Affiliations:** 1 Universidade Federal de Santa Maria, Departamento de Enfermagem, Santa Maria, RS, Brazil.; 2 Universidade Franciscana, Centro de Ciências da Saúde, Santa Maria, RS, Brazil.; 4 Pontifícia Universidade Católica do Rio Grande do Sul, Escola de Ciências da Saúde e da Vida, Porto Alegre, RS, Brazil.; 5 Universidade Federal de Santa Maria, Santa Maria, RS, Brazil.

**Keywords:** Patient Safety, Organizational Culture, Quality of Health Care, Nursing Care, Obstetric Nursing, Cross-Sectional Studies, Segurança do Paciente, Cultura Organizacional, Qualidade da Assistência à Saúde, Cuidados de Enfermagem, Enfermagem Obstétrica, Estudos Transversais, Seguridad del Paciente, Cultura Organizacional, Calidad de la Atención de Salud, Atención de Enfermería, Enfermería Obstétrica, Estudios Transversales

## Abstract

**Objective:**

to assess the correlations between the patient safety culture, the missed Nursing care, and the reasons for the omission in the obstetric area.

**Method:**

a cross-sectional study, conducted in 2019, with 62 Nursing professionals working in the obstetric area of a teaching hospital in southern Brazil. The MISSCARE-Brasil and Hospital Survey on Patient Safety Culture instruments were used. The data were analyzed using descriptive statistics, means comparison test and Spearman correlation.

**Results:**

the overall mean of positive answers for the safety culture was 34.9 (± 17.4). The care of assessing the vital signs and monitoring capillary blood glucose were the most prioritized, with airway aspiration and oral hygiene being the most overlooked. The main reasons for the omissions refer to labor resources and to inadequate staffing. A significant and inversely proportional correlation was found between the patient safety culture and overlooked nursing care (r=-0.393).

**Conclusion:**

the safety culture of the obstetric area was assessed as fragile by the Nursing professionals. The more the safety culture is strengthened and the greater investment in labor and human resources, the less care is overlooked.

## Abstract (pt)

**Objetivo:**

avaliar as correlações entre a cultura de segurança do paciente, os cuidados de enfermagem omitidos e as razões da omissão na área obstétrica.

**Método:**

estudo transversal, realizado em 2019, com 62 profissionais da enfermagem atuantes na área obstétrica de um hospital de ensino do Sul do Brasil. Utilizaram-se os instrumentos MISSCARE-Brasil e *Hospital Survey on Patient Safety Culture*. Os dados foram analisados por meio de estatística descritiva, teste de comparação de médias e correlação de Spearman.

**Resultados:**

a média geral de respostas positivas para a cultura de segurança foi de 34,9 (± 17,4). Os cuidados de avaliação dos sinais vitais e monitoramento da glicemia capilar foram os mais priorizados, sendo a aspiração de vias aéreas e higiene bucal os mais omitidos. As principais razões para as omissões referem-se aos recursos laborais e ao número inadequado de pessoal. Constatou-se correlação significativa e inversamente proporcional entre cultura de segurança do paciente e cuidados de enfermagem omitidos (r = -0,393).

**Conclusão:**

a cultura de segurança da área obstétrica foi avaliada pelos profissionais da enfermagem como frágil. Quanto mais fortalecida a cultura de segurança e maior investimento nos recursos laborais e humanos, menor o número de cuidados omitidos.

## Abstract (es)

**Objetivo:**

evaluar las correlaciones entre la cultura de seguridad del paciente, los cuidados de enfermería omitidos y las razones de la omisión en el área obstétrica.

**Método:**

estudio transversal, realizado en 2019, con 62 profesionales de enfermería que trabajan en el área obstétrica de un hospital universitario del sur de Brasil. Se utilizaron los instrumentos MISSCARE-Brasil y *Hospital Survey on Patient Safety Culture*. Los datos se analizaron mediante estadística descriptiva, prueba de comparación de medias y correlación de Spearman.

**Resultados:**

la media general de respuestas positivas para la cultura de seguridad fue 34,9 (± 17,4). El cuidado de la evaluación de los signos vitales y la monitorización de la glucemia capilar fueron los más priorizados, siendo la aspiración de las vías respiratorias y la higiene bucal los más omitidos. Las principales razones de las omisiones se deben a los recursos laborales y al número inadecuado de personal. Se constató una correlación significativa e inversamente proporcional entre la cultura de seguridad del paciente y los cuidados de enfermería omitidos (r = -0,393).

**Conclusión:**

la cultura de seguridad del área obstétrica fue evaluada por profesionales de enfermería como débil. Cuanto más se fortalece la cultura de seguridad y más se invierte en mano de obra y recursos humanos, menos cuidados se omiten.

## Introduction

As one of the dimensions of quality of care, patient safety is of increasing importance for patients, families, professionals and health managers. In Nursing, the increase in this visibility reflects the constant search for care based on scientific evidence and congruent with the context of care provision^(^ 1 ^)^.

In Brazil, this greater visibility occurred with the commitment established in 2004 with the World Alliance for Patient Safety and the institution of the National Patient Safety Program, implemented in 2013, by Decree No. 529/2013. This aims to contribute to the qualification of care in all health sectors, through the implementation of initiatives aimed at patient safety^(^ 2 ^)^.

However, there are still challenges in promoting patient safety. A health incident is estimated to occur every 35 seconds. In developing countries, several factors are unfavorable to patient safety^(^ 3 ^)^. Among them, overcrowding, deficit of professionals, inadequate infrastructure, and poor sanitary and hygiene conditions stand out^(^ 3 ^)^. The lack of Nursing care is also an unfavorable aspect^(^ 4 ^)^, being considered as an omission error^(^ 5 ^)^. Omission of care is defined as any patient need that has not been fulfilled (partially or totally) or that has been met with significant delay^(^ 5 ^)^.

The Nursing care missed, as well as the reasons for this omission, have been studied in different contexts^(^ 4 ^-^ 8 ^)^, presenting different justifications, such as the imbalance between excessive demands and insufficient resources^(^ 6 ^)^, which hinder compliance with the care prescribed. In such a situation, the professionals need to establish priorities and are often unable to perform all the care^(^ 6 ^,^ 9 ^)^. The Missed Nursing care model seeks to explain this phenomenon and assumes that institutional specificities (type of hospital, unit and characteristics of the workers) exert an influence on the Nursing work process, with the possibility of leading to professional dissatisfaction and harms to the patients^(^ 5 ^)^.

The work process of the Nursing team is structured around caring for or assisting, managing or administering, teaching and researching. It develops in systematized activities that aim at providing comprehensive health care to the population. The reorganization of the Nursing work process is a constant challenge to comply with safety standards and guarantee quality of care. Within the safety culture, the category of Nursing professionals has a fundamental role, due to the greater involvement in hospital processes and the greater proximity to the patients, being responsible for the quality of care they provide^(^ 8 ^)^.

In the obstetric area, missed care can have a negative impact on quality of care and on the safety of both women and newborns^(^ 7 ^,^ 10 ^)^. Better care before and after delivery could prevent 1.49 million maternal and neonatal deaths each year in the world^(^ 11 ^)^. Regarding omission of care, there is evidence that investing in hiring obstetric nurses causes harmonization in the administration and optimization of the Nursing services^(^ 7 ^)^. The organization of the work shifts and the satisfaction of the professionals can minimize omissions^(^ 7 ^)^, as well as the use of patient safety protocols and checklists, like the Safe Childbirth Checklist (SCC), which favors the standardization of essential care for delivery and birth and the development of a patient safety culture^(^ 12 ^)^.

According to the literature, the lack of Nursing care is proportional to the lack of patient safety^(^ 4 ^)^. A study carried out in Turkey verified that midwifery professionals would report fewer Nursing care omissions compared to surgical and rehabilitation units^(^ 13 ^)^. In this context, when identifying the care omitted, information is obtained that can be used by the service management to improve quality and safety in care^(^ 9 ^)^.

In the obstetric context, studies were found that individually assess both omission of care and its reason^(^ 7 ^)^, regarding the safety culture^(^ 14 ^)^. The analysis of the relationship between these objects of investigation is important and necessary, as it can assist in obtaining new knowledge and in developing attitudes that promote a positive culture^(^ 9 ^)^, in addition to subsidizing professionals and managers in the construction of a collaborative care model and, consequently, of a stronger patient safety culture.

In this study, the objective was to assess the correlation between the patient safety culture, the missed Nursing care and its reasons in the obstetric area. It has been hypothesized that the more the safety culture is strengthened, the less care is omitted and there are fewer reasons for its omission.

## Method

This is a cross-sectional study, with a quantitative approach.

It was carried out at the Obstetric Center (OC) and in the Rooming-In (RI) of the Women’s Health Care Unit (WHCU) of a teaching hospital in the central region of Rio Grande do Sul (RS), Brazil. This is a general hospital, of medium and high complexity, with exclusive care by the Unified Health System (*Sistema Único de Saúde*, SUS). Thus, the choice for this service was due to being a high-complexity reference in the care of high-risk pregnancies. It works as an obstetric emergency service for 45 municipalities, serving approximately 700 consultations and 200 deliveries/month, by a multi-professional team.

The research was carried out from August 21st to November 13^th^, 2019.

All the Nursing professionals (nurses, technicians and assistants) working for at least 30 days in the units surveyed were included. Professionals who were away from work for any reason during data collection were excluded. Thus, the population was composed of 85 professionals. Of these, 17 (20%) were excluded for not meeting the selection criteria. Thus, the eligible population was 68 (100%) professionals. Of these, six (8.8%) constituted losses (refusal to participate and non-return of the instruments). 62 (91.2%) workers participated in the study, composing a sample for convenience and non-probabilistic.

The independent variables of the study were composed by sociodemographic (gender, age, schooling level), work (time working at the hospital, number of hours worked, function/position, work shift) and level of patient safety culture. The dependent variables were the missed Nursing care and the reason for the care omitted.

Two instruments were used, translated and validated for the Brazilian culture, namely: MISSCARE-Brasil^(^ 9 ^)^ and the Hospital Survey on Patient Safety Culture (HSOPSC)^(^ 15 ^)^, in printed format. These instruments have already been used in research in the obstetric area^(^ 7 ^,^ 13 ^-^ 14 ^,^ 16).

The HSOPSC questionnaire^(^ 15 ^)^ is used to capture and measure the perception of professionals regarding the multiple dimensions of the patient safety culture. It contains 42 items, covering 12 dimensions of the safety culture assessed at the individual, unit and hospital levels. The instrument has questions answered on a five-point Likert scale, ranging from “I strongly disagree” to “I strongly agree”; or from “never” to “always”. The items are constructed positively, where the concordant answer is positive for the safety culture, as well as there are items written negatively, whose dissenting answer is the positive one^(^ 15 ^)^.

The MISSCARE-Brasil^(^ 9 ^)^ instrument consists of two parts. The first has 28 items referring to the elements of Nursing care not performed, with Likert-type answers, ranging from “it is never performed” to “it is always performed”. Items are scored from 1 to 5, where 1 corresponds to the highest levels of omission and 5 to the absence of omission. The second part of the questionnaire also contains 28 items consistent with the reasons for not performing Nursing care, with answers on a four-point Likert scale, ranging from 1 – “significant reason” to 4 – “it is not a reason for the omission of care”. The reasons for not performing Nursing care are divided into five categories: communication; material resources; labor resources; ethical dimension; and institutional management/leadership style^(^ 9 ^)^. Authorization and guidance from the authors were obtained for the use of the instrument.

The data were collected in the workplace, during the morning, afternoon, and night shifts, by two collectors (graduating and graduate students) previously trained by the responsible researcher. The instruments are self-administered and the completion time for all questions was approximately 20 minutes. As they were validated and widely used questionnaires, no pilot test was performed.

The data entry process was performed with independent double entry, by previously trained typists. Errors and inconsistencies were verified and corrected by the researcher in the review process, using the “validate” feature of the Epi-Info software® (version 6.4). Afterwards, data analysis was performed using the SPSS (Statistical Package for the Social Sciences, SPSS Inc., Chicago) program, version 18.0 for Windows.

The reliability of the instruments was tested by analyzing the internal consistency, using Cronbach’s alpha coefficient. Values > 0.70 were considered indicative of internal consistency. Data normality was assessed using the Shapiro-Wilk test^(^ 17 ^)^.

The categorical variables were assessed using absolute (N) and relative (%) frequencies; and the quantitative variables, by mean, median, standard deviation, minimum, and maximum, according to the normality or not of the data. The test of means comparison (Mann-Whitney’s U Test) was carried out between professional category, safety culture, missed Nursing care and the reasons for the omission. Spearman correlation analyses were also carried out, adopting the following points: r from 0.10 to 0.39 as a weak dependence between the variables; from 0.40 to 0.69 as moderate; and from 0.70 to 1.00 as a strong correlation^(^ 18 ^)^. In all the analyses, a significance level of 5% (p<0.05) was used.

The analysis and interpretation of the HSOPSC results followed the authors’ recommendations^(^ 15 ^)^. The scale was recoded from five to three levels, where 1 corresponds to the total of negative answers, 2 corresponds to neutral answers, and 3 relates to positive answers (adding points 4 and 5). The percentage of positive answers represents a positive reaction to the patient safety culture and allows identifying strong and fragile areas in patient safety. “Strong areas of the patient safety culture” are those whose items obtained 75% or more of positive answers. On the other hand, if the mean percentage of positive answers is equal to or less than 50%, it is understood that this is a fragile area, suggesting a culture with negative aspects and in need of improvement.

For the MISSCARE-Brazil scale, a descriptive analysis was performed, with simple frequency and grouping of the answer options, “frequently” and “are always performed”^(^ 9 ^)^. The prevalence of omission of each care action was calculated by dividing the number of care actions omitted by the total amount of answers obtained by that element of Nursing, multiplied by 100. Likewise, the prevalence of the reasons for omission was calculated by dividing the number of answers considered as a reason for omission by the total number of answers obtained by that reason, multiplied by 100.

This research was approved the institution’s Research Ethics Committee, via *Plataforma Brasil* (Opinion No. 3,470,447). All recommendations of the National Health Council (Resolution 466/2012) for research with human beings were followed. The professionals who participated in the study were invited on duty shifts and individual approaches, and they received, read, and signed the Free and Informed Consent Term, in two copies, on the same day they answered the questionnaire.

## Results

The survey included women (100%), with a mean age of 39.1 years old (± 10.1), ranging from 19 to 62 years old. Of these, 61% (N=38) were nursing technicians and assistants and 39% (N=24) were nurses, working in the RI area (53.2%; N=33) and in the OC (46.8%; N=29). They have been worked in the institution for less than 10 years (63%; N=39), ranging from four months to 28 years. Night work predominated (37%; N=39), as well as a weekly workload of over 30 hours (71%; N=44), ranging from 44 to 60 hours and daily 6-hour shifts (48%; N=30). In a higher percentage, they had postgraduate degrees (53.2%; N=33), 27.4% (N=17) in the obstetric area.

Regarding the adequacy of the number of professionals in the work schedule, 3.3% (N=2) stated that it was adequate and 16% (N=10) said it was inadequate. Regarding the number of patients cared for in the last shift, 50% (N=31) of the professionals answered that they had provided assistance to 10 (2-28) patients, including women and newborns. The number of admissions presented a median of 3 (0-11), and the number of discharges a median of 1 (0-8).

Regarding satisfaction, a high level was identified in relation to the profession and position (89%; N=55), and with the unit (85%; N=53). In contrast, 47% (N=29) were, in an intermediate way, satisfied with teamwork. When asked about plans to leave the job or the position they held, 6.6% (N=4) of the workers answered affirmatively. As for patient safety in the obstetric area, more than half of the workers (61%; N=38) considered it to be regular. For 58% (N=36), there were no adverse event notifications in the last 12 months.

The internal consistency of the HSOPSC and MISSCARE-Brasil instruments was adequate (α=0.87 and α=0.89, respectively). In Table 1, the dimensions of the patient safety culture in the hospital’s obstetric area will be presented, regarding the percentage of positive answers.

**Table 1:** Percentage of positive answers, in the obstetric area, of the general safety culture and according to the 12 dimensions of the Hospital Survey on Patient Safety Culture. Santa Maria, RS, Brazil, 2019 (N=62)

Dimensions of the HSOPSC*	% of answers positive
Patient Safety Culture in the units	34.9
Organizational learning	56.9
Expectations of supervisors' safety promotion actions	50.0
Teamwork in the unit	43.1
Frequency of notified events	43.0
Opening for communication	40.0
Changeover of internal transfers	37.2
Teamwork between hospital units	31.8
Feedback of information communication about errors	31.5
General Perception of Patient Safety	27.2
Professionals/Staffing	25.9
Hospital management support for PS	23.6
Non-punitive responses to errors	9.1

* HSOPSC = Hospital Survey on Patient Safety Culture

According to Table 1, none of the dimensions was assessed as strong for the safety culture by the professionals. The best percentage of positive answers was obtained in the following dimensions: organizational learning (56.9%) and expectations of supervisors’ safety promotion (50%), which can be considered as intermediate. The non-punitive responses to errors dimension presented the lowest levels (9.1%). The overall mean of positive answers for the general patient safety culture in the unit was 34.9 (± 17.4).

As for Nursing care in the obstetric area, the distribution of answers by frequency with which each element of care was performed is shown in Table 2.

**Table 2:** Description of the frequency (%) of Nursing care according to the grouping of occasionally, frequently and always performed options. Santa Maria, RS, Brazil, 2019 (N=62)

Nursing Care	Often/Always performed	
	N	%
Assessment of vital signs as prescribed.	61	98.4
Capillary blood glucose monitoring as prescribed.	59	95.2
Assessment of the patient's condition at each shift, identifying their care needs.	56	90.3
Focused reassessment, according to the patient's condition.	55	88.8
Care with venous access and infusion, according to the institution's rules.	55	88.8
Hand hygiene.	55	88.8
Assessment of the effectiveness of the drugs administered.	53	85.5
Complete record in the patient's medical record of all the necessary data.	51	82.3
Emotional support to the patient and/or family.	51	82.3
Guidelines to patients and family members regarding routines, procedures and care provided.	49	79.0
Requests for administration of prescription drugs W/N are met in fifteen minutes.	48	77.4
Use of preventive measures for patients at risk of falling.	47	75.8
Planning and teaching the patient and/or family for hospital discharge.	47	75.8
Bathing/Patient hygiene/Measures to prevent skin injuries.	46	74.2
Administration of the medications within 30 minutes before or after the prescribed time.	46	74.1
Hydration of the patient, when adequate, offering fluids orally or administering through the tube.	45	72.6
Care of skin lesions/wounds.	44	70.9
The answer to the patient's call is within five minutes.	44	70.9
Offering meals to the patients who eat by themselves.	44	70.9
Walking three times a day or as prescribed.	42	66.7
Water balance control: inputs and outputs.	40	64.5
Sitting the patient out of bed.	40	64.6
Participation in discussion of the interdisciplinary team on patient care, if it occurs.	33	53.2
Sanitizing the patient promptly after each elimination.	31	50.0
Feeding the patient or administering the diet by tube, on time.	31	50.0
Changing the patient's position every two hours.	30	48.4
Airway aspiration.	27	43.6
Oral hygiene.	26	42.0

According to Table 2, assessing the vital signs and monitoring capillary blood glucose were the most prioritized activities (98.4% and 95.2%, respectively), followed by the assessment of the patient’s conditions at each shift, identifying their care needs (90.3%); focused reassessment, care with venous access and hand hygiene (88.8% each). The most omitted care actions were related to airway aspiration (43.6%) and oral hygiene (42.0%).

The Nursing team’s understanding of the reasons for not providing care is presented by domains in Table 3, according to MISSCARE-Brazil.

**Table 3:** Frequency (%) distribution of the answers, by category of reasons for omitting Nursing care according to the grouping of the significant and moderate options. Santa Maria, RS, Brazil, 2019 (N=62)

Reasons for omitting care	Significant ratio/ Moderate ratio	
**Labor resources**	**N**	**%**
Inadequate staffing.	53	85.4
Inadequate staffing for assistance or administrative tasks (e.g., inadequate number of clerks/secretaries, nursing assistants, technicians or nurses).	50	80.7
Large number of admissions and discharges.	43	69.3
Unexpected increase in volume and/or severity of the patients in the unit.	41	31.1
Emergency situations of the patients (e.g.: worsening of a patient's condition).	40	64.6
High number of professionals who work sick or with health problems (which prevents them from performing the functions for which they were hired).	36	58.1
High number of nurses with little professional experience.	25	40.3
The professionals have more than one job, which reduces their commitment/attention/concentration to perform the assistance.	18	29.0
**Material resources**		
The medications were not available when needed.	39	62.9
The physical plant of the unit/sector is inadequate, which makes it difficult to provide assistance to patients in isolation or in more distant areas.	38	61.3
The materials/equipment did not work properly when needed.	35	56.4
The materials/equipment were not available when needed.	33	53.2
**Communication**		
The distribution of patients by professional is not balanced.	33	53.2
Tension /Conflict or communication problems within the Nursing Team.	33	53.2
Tension/Conflict or communication problems with the Medical Team.	33	53.2
The nursing assistant did not report that the assistance was not performed.	33	53.2
Tension/Conflict or problems communicating with other departments/support sectors.	30	48.4
The professional responsible for the care was outside the unit/sector or was not available.	27	43.6
Other team professionals did not provide assistance at the time it was needed (e.g.: the physical therapist did not assist in the patient's walking).	26	42.0
The team members do not help each other.	26	42.0
Lack of standardization for performing procedures/care actions.	24	38.7
The changeover from the previous shift or from the units that refer patients is inappropriate.	23	37.1
**Ethical dimension**		
The professional has no ethical posture and has no commitment and involvement with the work and/or the institution.	29	46.8
The professional who did not provide care is not afraid of punishment/dismissal due to job stability.	27	43.5
The Nursing professional is negligent (laziness, lack of attention or insensitivity).	24	38.7
**Institutional Management/Leadership**		
Lack of preparation of nurses to lead, supervise and conduct teamwork.	36	58.1
Lack of in-service education about the care to be performed (which includes training, updating, improvement and professional development).	29	46.8
Lack of motivation for work (due to low wages and/or lack of professional appreciation).	25	40.3

Labor resources were the main reason for omitting or postponing Nursing care, followed by material resources. In these categories, the main reasons were related to inadequate staffing (85.4%), followed by the large number of admissions and discharges (69.3%) and the emergency situation of the patients (64.6%). The professionals having more than one job was not considered a reason to reduce their commitment/attention/concentration to perform the assistance (29%).

Table 4 shows the means comparison between the professional categories of the Nursing team (Nurses and Nursing technicians/assistants), safety culture, missed Nursing care and the reasons for omission in Obstetrics.

**Table 4:** Means comparison between the professional categories and patient safety culture, missed Nursing care and reasons for omission in the obstetric area. Santa Maria, RS, Brazil, 2019 (N=62)

Variables	Nurses				Nursing technicians and assistants				p*
	Mean (LL-UL)	SD^‡^	Median	Min-Max^§^	Mean (LL-UL)	SD	Median	Min-Max	
HSOPSC^||^	27.0 (19.9-34.2)	16.1	24.6	2.1-58.3	39.8 (33.4-46.3)	17.2	40.6	4.9-72.9	0.712
Missed Nursing care	47.1 (42.7-51.4)	10.4	45.4	27.9-69.3	36.9 (33.1-40.6)	10.6	37.1	20.7-67.1	0.702
Reasons for omission	64.2 (58.6-69.8)	13.3	63.8	42.0-86.6	64.0 (58.1-69.9)	16.6	67.9	25.9-89.2	0.416
Communication	2.4 (2.2-2.7)	0.5	2.4	1.4-3.4	2.3 (2.1-2.6)	0.7	2.5	1.0-3.5	0.107
Material resources	2.7 (2.5-3.0)	0.7	2.7	1.0-3.7	2.8 (2.5-3.0)	0.7	2.7	2.71,0-4.0	0.742
Labor resources	2.7 (2.5-3.0)	0.5	2.7	7.9-3.5	2.8 (2.6-3.0)	0.7	3.0	1.0-3.7	0.526
Ethics	2.4 (1.9-2.8)	1.0	2.2	1.0-4.0	2.4 (2.1-2.8)	1.0	2.3	1.0-4.0	0.996
Institutional Management/ Leadership	2.4 (0.1-2.8)	0.8	2.5	1.0-4.0	2.5 (2.1-2.8)	1.0	2.7	1.0-4.0	0.171

* Mann-Whitney's U Test;

†LL-UP = Lower Limit - Upper Limit;

‡ SD = Standard Deviation;

§ Min-Max = Minimum - Maximum;

|| HSOPSC = Hospital Survey on Patient Safety Culture

According to Table 4, there was no statistically significant difference between the professional categories and the safety culture, missed Nursing care and reasons for the omission (p>0.05).

Figure 1 shows the correlations between patient safety culture, missed Nursing care and reasons for the omission of care perceived by the professionals of the Nursing team in the obstetric area.

**Figure 1:** Correlations of the Safety Culture and its dimensions with the missed Nursing care score, reasons for the omission and its categories in the context of Obstetrics. Santa Maria, RS, Brazil, 2019 (N=62)

Variables	1	2	3	4	5	6	7	8	9	10	11	12	13	14	15	16	17	18	19	20
1. Hospital Survey on Patient Safety Culture	1																			
2. Teamwork between hospital units	.657^†^	1																		
3. Expectations of supervisors' safety promotion actions	.638^†^	.436^†^	1																	
4. Organizalional leaming	.731^†^	.496t	466^†^	1																
5. feedback of Information communication about errors	.571^†^	.350	.451^†^	.414^†^	1															
6. Opening for communication	.731^†^	.471 ^†^	^†^.576^†^	.453^†^	.555*	1														
7. Prfessionals/Staffing	-.088	.186	^†^.-.064	.-.027	-.045	-0.71	1													
8. Non-punitive responses to error	.268*	.257*	.222	.205	.085	.082	-0.11	1												
9. Hospital management support for PS	.608^†^	.3581	.512^†^	.509t	.311*	.349^†^	-.005	.107	1											
10. Teamwork in the unit	.700t	.341t	-236	.460t	.362^†^	.548^†^	-134	.183	.405^†^	1										
11. Changeover of internai transfers	.648t	.396	^†^.282*	.393^†^	.297*	.417^†^	-.084	.272*	.304*	.453^†^	1									
12. General Perception of pateint Safety	.572^†^	361	^†^.281*	.562^†^	.448^†^	.292*	.109	.108	.563^†^	.495^†^	.377^†^	1								
13.frequency of notified events	.356^†^	.074	.018	.190	.119	.039	-.045	-.034	.128	.235	.179	.207	1							
14. Missed Nursing care	-393^†^	-.238	-.271*	-519^†^	-.191	-.223	-.185	-.120	-.332^†^	-.277*	-.323*	-.366^†^	-.2351	1						
15. Reasons for omission	-.192	-.117	-.222	-238	-.132	-.238	.009	.077	-.158	-.078	-.100	-.006	.076	.257*	1					
16. Communication category	-.224	-.230	-.260*	-.192	-.157	-.263*	-.034	.068	-.215	-.095	-.072	-.012	.040	.311*	921^†^	1				
17. Material resources category	-.289	-.118	-.265*	-.170	-.141	-.249	0.23	-.100	-.124	-.178	-.189	-.069	-.103	.093	.685^†^	.616^†^	11			
18. Labor resources category	-.109	-.046	-.119	-.160	-.064	-.200	-.061	.071	-.074	-.059	-.172	-.098	.120	.157	.803^†^	.618^†^	.565^†^	1		
19. Ethical category	59	-.044	-.110	-.250	-.123	-.117	.051	.063	-.121	-.002	.063	.030	.082	.218	.836^†^	.750^†^	.442^†^	.522^†^	1	
20. Institucional Management/Leadership category	-.004	.029	-.142	-.172	-.022	-.133	.083	.234	-.089	.080	-.086	.080	-.0.86	.254*	.810^†^	.682^†^	.376^†^	.666^†^	.667^†^	1

* The correlation is significant at the 0.05 level; to 1.00) correlation^(^ 18 ^)^

† The correlation is significant at the 0.01 level. Weak (0.10 to 0.39), moderate (0.40 to 0.69) and strong (0.70

Strong (r=0.7 to 1.0), significant and direct correlations were identified between the general safety culture and the domains of organizational learning, open communication and teamwork in the unit; moderate (r=0.4 to 0.69), significant and direct correlations with the domains of teamwork between units, expectations of supervisors’ safety promotion actions, feedback, management support, shift change for internal transfers and general perception of safety; weak (r=0.1 to 0.39), significant and direct correlations with the non-punitive responses to the errors and frequency of errors notified domains.

There was a weak, significant and inversely proportional correlation between the general patient safety culture, missed Nursing care (r=-0.393) and the material resources category for the reason of the omission (r=0.289). In other words, the more the safety culture is strengthened, the smaller the number of omissions in care.

Regarding the omitted care, a significant, weak and inverse correlation was obtained with the domains of safety culture: expectations of supervisors’ safety promotion actions, organizational learning, management support, teamwork, shift change, and general perception of patient safety. There was a significant, weak and direct correlation between the reasons for the omission and the missed Nursing care. In the same way, significant, strong and direct correlations were identified between reasons for omission and communication, material and labor resources, ethics and management/leadership.

## Discussion

Predominance of women was observed in this study. This global phenomenon in the health sector is related to female professionalization, which has been strongly linked to gender roles in society. Furthermore, in the maternal and neonatal area, historically, care was provided by, for and with women, full of meanings and empowerment^(^ 19 ^)^.

The weekly workload of midwifery professionals is an aspect that needs to be discussed. The development of excessive working hours can alter the physical and psychological functioning of the worker and, consequently, negatively influence the provision of safe care^(^ 16 ^)^. A study carried out in Finnish hospitals concluded that the workload of Nursing professionals above the level considered adequate can increase between 8% and 34% the chances of safety incidents and adverse events occurring^(^ 20 ^)^.

A low perception of positive answers about the patient safety culture was identified by the Obstetric Nursing professionals, presenting more fragile than strengthened dimensions for the safety culture. These findings are similar to those of a research study carried out in three Brazilian maternity hospitals, with a general culture score of 40.7%. In the same study, of the 12 dimensions, nine had scores below 50%^(^ 16 ^)^. In the investigation of weaknesses and potentialities regarding the safety culture, it is possible to highlight patterns of behavior and actions with a view to improving the quality of care provided, providing subsidies to seek more positive results^(^ 20 ^)^.

A better percentage of positive answers was obtained in the dimensions of organizational learning and supervisors’ expectations of safety promotion. The first assesses the existence of learning from the errors that lead to positive changes and the effectiveness of the changes that have occurred; the second analyzes whether supervisors and managers consider the professionals’ suggestions to improve safety, recognizing and encouraging their participation in the improvements^(^ 15 ^)^.

This data was similar to a study developed in a general hospital^(^ 21 ^)^ and differs from a survey carried out in three maternity hospitals that considered these dimensions to be the most worrying, as they presented a lower percentage^(^ 16 ^)^. Thus, the importance of the involvement and performance of the leaders in order to provide safe care is evidenced, based on educational lessons learned and shared among the team, through the errors reported^(^ 22 ^)^.

In this study, the most fragile dimension was *non-punitive responses to errors*, indicating the existence of a culture of culpability, which blames the professional, disregarding the systemic factors involved in the occurrence of an error. In the units surveyed, the professionals believe that their errors can be used against them. This shows that, despite the efforts of the managers, strategies are still needed to encourage learning from errors Identifying and improving flaws in the work process, based on dialog, active and sensitive listening, stimulating and welcoming the needs of workers and encouraging them to notify^(^ 22 ^)^.

When analyzing the most performed Nursing care actions, the following stand out: assessment of vital signs and monitoring of capillary blood glucose; as well as assessment of the patient’s conditions at each shift, identifying their care needs, focused reassessment and care with venous access and infusion. Such care actions can be linked to the specificity of the obstetric area, as they are reference units for high-risk pregnant women, where there are well-worked protocols and guidelines with professionals for monitoring patients that can quickly destabilize. In addition to that, they favor the reduction of the main causes of maternal deaths, such as: hemorrhage, infections, pre-eclampsia and eclampsia^(^ 10 ^)^.

The most neglected Nursing care measure were airways aspiration every two hours and oral hygiene, this data can be understood due to the singularities of the area. That is, for pregnant women and newborns hospitalized in the OC or RI, there are hardly any prescriptions for these Nursing care actions. However, this finding is consistent with the international literature, regarding the care most omitted by Nursing^(^ 8 ^)^.

From the use of the MISSCARE-Brazil instrument, the professionals related the care they performed the least with the reasons. It was verified that inadequate staffing and the large number of admissions and discharges, followed by lack of material resources were the main causes of omissions. These data are consistent with a research study that used the same instrument^(^ 23 ^)^, with inadequacy of people being one of the most debated issues in the health area, understood as a way to ensure more effective care and safety. A study carried out in Ethiopia on missed care in the maternity hospital confirms the need for more nurses in perinatal care and the adequacy of material resources^(^ 7 ^)^.

Despite the uniqueness of the Nursing team’s work process, between the categories of mid-level and higher level professionals, in this study there was no statistically significant difference between them and the missed care, the reasons for omission and the safety culture. In contrast, a study conducted in São Paulo identified that the nurses reported more reasons for omitting care when compared to nursing technicians, disagreeing in all the domains (p<0.05), except in the communication domain (p=0.08)^(^ 6 ^)^.

The hypothesis of this study was confirmed, showing an inversely proportional correlation between the patient’s safety culture and missed Nursing care. This data reinforces a research study^(^ 24 ^)^ that indicates that the more strengthened the safety culture of an institution, the lower the omission of care. Inverse correlations were observed between missed Nursing care in the obstetric area and some dimensions of the safety culture. These demonstrate the importance of the management’s commitment to prioritizing multidisciplinary and transversal work on patient safety in all care contexts and levels. Such a measure helps in minimizing missed Nursing care and, therefore, the adverse results to the patients^(^ 24 ^)^.

The results of this study confirm the importance of communication for the safety culture and for the reduction of the reasons for omission. It was observed that the more available the professionals are to communication, the less the possibility of care being omitted. Thus, an institutional organization accessible to the dialog on safety proposes freedom for the professionals to identify and prevent problems that could result in missed or delayed care. It is believed that a management committed to promoting safety facilitates communication among the team members.

In this context, the findings that the investment in material resources (medications, equipment and infrastructure, among others) available and in operation for the team reduces the reasons for the omission of care and highlights the management’s concern with promoting a safety culture. This result confirms that the better the practice environment, the lower the volume of care that is left to do^(^ 25 ^)^.

In the obstetric area, this view is important, considering the vulnerability of women and families in the puerperal pregnancy process, when, for example: they have no privacy; they need to stay on stretcher labor; when they are not offered non-pharmacological methods of pain relief^(^ 26 ^)^, making it necessary to use tools to ensure more effective care. Thus, investing in a suitable environment to accommodate the needs of pregnant women and parturients, such as investing in individual pre-delivery, delivery and post-delivery rooms, adequate lighting, bathtubs, balls and space to walk, among others, is a strengthening of the safety culture, as well as it humanizes and prevents the omission of Nursing care.

The limitations of this study are understood to be the restricted number of participants and the specific context. However, its importance is also at this point, due to the possibility of making a situational diagnosis correlating these themes. It is noteworthy that this panorama is essential to sensitize professionals and managers about these constructs, helping them to understand the singularities of the area, considering that they are recent discussions and full of stigmas that need to be relaxed by the professionals and by the management. It is recommended to carry out mixed-methods studies, which allow for greater immersion with the professionals, so that improvement strategies are identified both for the safety culture and for the prevention of care omissions.

The relevance of this study for the professional practice is to identify that the reasons, related to the lack of labor and material resources and to failures in obstetric communication can lead to the occurrence of the Nursing care omissions. As well as understanding that, for the strengthening of the safety culture, it is fundamental to stimulate discussions about the weakened work processes, understanding error as an opportunity for improvement and learning. With this, enhancing and guaranteeing the quality of Nursing care for women and newborns.

## Conclusion

The safety culture of the obstetric area was assessed as fragile by the Nursing professionals. Care for monitoring capillary blood glucose and assessing vital signs were the most prioritized, while changing the position and feeding of patients were the most overlooked. The main reasons for the omissions refer to labor resources and to inadequate staffing. There was no statistically significant difference between the professional categories and the safety culture, missed care and reasons for the omission.

However, a significant and inversely proportional correlation was found between patient safety culture and missed Nursing care. Confirming the study hypothesis, it became evident that the stronger the safety culture, the less Nursing care will be omitted.
